# Thomas, Medical Dictionary

**Published:** 1885-12

**Authors:** 


**Thomas' Medical Dictionary.** A complete pronouncing medical dictionary, embracing the terminology of medicine and the kindred sciences, with their signification, etymology, and pronunciation. With an appendix comprising an explanation of the Latin terms and phrases occurring in medicine, anatomy, pharmacy, etc; together with the necessary directions for writing Latin prescriptions, etc., etc. By JOSEPH THOMAS, M. D., L. L. D. Philadelphia: J. B. Lippincott Company. 1886.

This work is designed for the medical student, as it "contains fuller explanations in regard to several subjects than has been usual in the preparation of medical dictionaries. One of the points to which especial attention has been given, is the etymology of the various terms occurring in the dictionary." In many respects, this work surpasses any now in use, and for the student we should say it is all the author claims for it. The style of the work is excellent and should have a large sale, not only to students, but to the practitioners throughout this country and England.

